# A Meta-Analysis about the Screening Role of Pulse Oximetry for Congenital Heart Disease

**DOI:** 10.1155/2017/2123918

**Published:** 2017-12-10

**Authors:** Caiju Du, Dianmei Liu, Guojing Liu, Huaixin Wang

**Affiliations:** ^1^Cardiovascular Surgery, Affiliated Hospital of Weifang Medical University, 2428 Yuhe Road, Kuiwen District, Weifang, Shandong Province 261031, China; ^2^Imaging Center, Affiliated Hospital of Weifang Medical University, 2428 Yuhe Road, Kuiwen District, Weifang, Shandong Province 261031, China; ^3^Operating Room, Affiliated Hospital of Weifang Medical University, 2428 Yuhe Road, Kuiwen District, Weifang, Shandong Province 261031, China; ^4^Emergency Department, Yidu Central Affiliated Hospital of Weifang Medical University, 4138 Linglong South Road, Qingzhou, Shandong Province 262550, China

## Abstract

**Objective:**

The opinions about the application of pulse oximetry in diagnosis of congenital heart disease (CHD) were debatable. We performed this meta-analysis to confirm the diagnostic role of pulse oximetry screening for CHD.

**Methods:**

Relevant articles were searched in the databases of Pubmed, Embase, Google Scholar, and Chinese National Knowledge Infrastructure (CNKI) up to April 2017. Data was processed in the MetaDiSc 1.4 software. Pooled sensitivity and specificity with 95% confidence interval (95% CI) were calculated to explain the diagnostic role of pulse oximetry screening for CHD. *I*^2^⩾50% or *p* < 0.05 indicated significant heterogeneity. Area under curve (AUC) of summary receiver operating characteristics (SROC) was calculated to assess its diagnostic accuracy. The robustness of overall results was evaluated by sensitivity analysis. Publication bias was evaluated by Deek's funnel plot.

**Results:**

22 eligible articles were selected. Pooled sensitivity and specificity were 0.69 (0.67–0.72) and 0.99 (0.99-0.99), respectively. The corresponding AUC was 0.9407, suggesting high diagnostic accuracy of pulse oximetry screening for CHD. Sensitivity analysis demonstrated that the pooled results were robust. Deek's funnel plot seemed to be symmetrical.

**Conclusions:**

Pulse oximetry screening could be used to diagnose CHD. It shows high diagnosis specificity and accuracy.

## 1. Introduction

Congenital heart disease (CHD) is regarded as a main cause of infant death, with an incidence of 8 in every 1000 live births [1]. It needs invasive intervention during the neonatal period and these neonates suffering this disease benefit most from early detection [2]. Prenatal diagnosis just picks up <50% of all cases [3–6]. Routine neonatal inspection fails to detect above than 50% of CHD infants. More than 55% of neonates show no murmur symptom in the nursery, and ⩽82% of them are discharged before diagnosis results are obtained [7]. Early diagnosis of CHD is crucial since the delayed diagnosis results in cardiovascular collapse, cardiac failure, and death, whereas early diagnosis during the first few days of life is difficult. Therefore, an effective screening program for CHD is necessary.

In recent years, pulse oximetry has been suggested as a diagnosis tool for CHD [8–10]. It is productive in the detection of CHD before discharge and could decrease missed cases to 4% [11, 12]. Some states of United States have made screening of pulse oximetry for CHD mandatory before discharge in hospital. Pulse oximetry can detect mild hypoxemia, which is common feature for many forms of CHD. It could recognize the cases that are not recognized by clinical examination [13]. Since the introduction of pulse oximetry to screen CHD in 1995, many studies have focused on the subject [14–17]. Despite the fact that there were differences in screening time, cut-off values, target lesions, and others among the relevant studies, the opinion is consistent that pulse oximetry screening is a useful diagnostic method of CHD. However, existing data is still insufficient to initiate a recommendation for application of pulse oximetry in routine care.

This present meta-analysis was performed to confirm the diagnostic role of pulse oximetry for CHD. The obtained results contribute to clinical application of pulse oximetry for diagnosing CHD.

## 2. Materials and Methods

### 2.1. Search Strategy

We searched the relevant articles on the databases of Pubmed, Embase, Google Scholar, and Chinese National Knowledge Infrastructure (CNKI) up to April 2017. The following keywords were used: pulse oximetry OR SpO2 AND congenital heart disease OR CHD. The references' lists of obtained articles were manually searched for eligible studies. No language restriction was applied. The studies published in abstract were not considered.

### 2.2. Article Selection

These obtained articles were selected according to inclusion criteria. The criteria were as follows: (a) SpO_2_ was assessed with pulse oximetry; (b) SpO_2_ was used to detect CHD subjects; (c) true positive (TP), false positive (FP), true negative (TN), and false negative (FN) or other data available for calculating them were reported. The review article, abstract article, and case reports were removed from the present analysis.

### 2.3. Data Extraction

Two authors were responsible for extracting data. The data included name of first author, year of publication, country, number of patients and healthy controls, screening time, screening limb, TP, FP, TN, and FN. The inconsistent opinion was solved with a discussion with the third author.

### 2.4. Statistical Analysis

Data was processed in the MetaDiSc 1.4 software. Deek's funnel plot was obtained with Stata 12.0 software. Summary sensitivity and specificity along with 95% confidence interval (95% CI) were adopted to confirm the diagnostic role of pulse oximetry screening for CHD. *I*^2^⩾50% or *p* < 0.05 indicated significant heterogeneity. Area under curve (AUC) of summary receiver operating characteristics (SROC) was calculated to assess the diagnostic accuracy of SpO_2_. The robustness of overall results was evaluated by sensitivity analysis. Publication bias was evaluated by Deek's funnel plot.

## 3. Results

### 3.1. Selection Process of Eligible Articles

**Figure 1:**
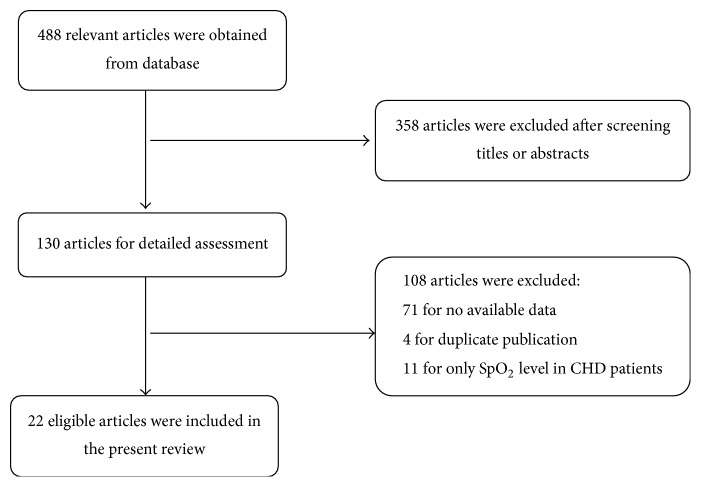
Flow chart of articles selection. 22 articles were selected for meta-analysis.

A total of 488 relevant articles were obtained after search on the databases. The titles and abstracts were screened and 358 articles were excluded. The remaining 130 articles were provided detailed assessment and 108 articles were excluded for no available data, duplicate publication, and only SpO_2_ level in CHD patients. Finally, 22 eligible articles were included in the present meta-analysis [8, 10–12, 14–31]. The detailed selection process was shown in Figure 1. Basic information of each study was listed in Table 1.

### 3.2. Diagnostic Role of Pulse Oximetry Screening for CHD

**Figure 2:**
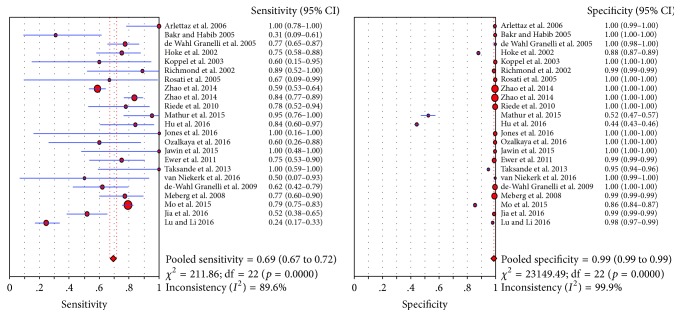
Diagnostic sensitivity and specificity of pulse oximetry screening for congenital heart disease (CHD). The sensitivity and specificity were 0.69 (0.67–0.72) and 0.99 (0.99-0.99), respectively.

**Figure 3:**
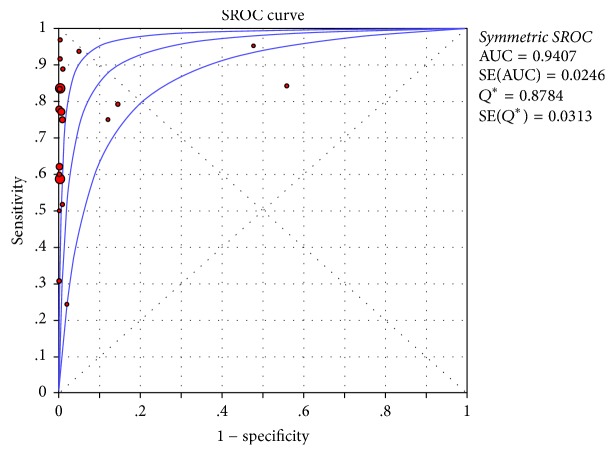
Area under curve (AUC) of SROC curve. AUC was 0.9407.

Pulse oximetry screening showed high specificity in detecting CHD (specificity: 0.99), while having relatively low sensitivity (0.69) (Figure 2). In the analyses of sensitivity and specificity, we observed significant heterogeneity (sensitivity: *p* = 0.0000, *I*^2^ = 89.6%; specificity: *p* = 0.0000, *I*^2^ = 99.9%). The corresponding AUC was 0.9407, suggesting high diagnostic accuracy of pulse oximetry screening for CHD (Figure 3).

### 3.3. Sensitivity Analysis and Publication Bias Detection

**Figure 4:**
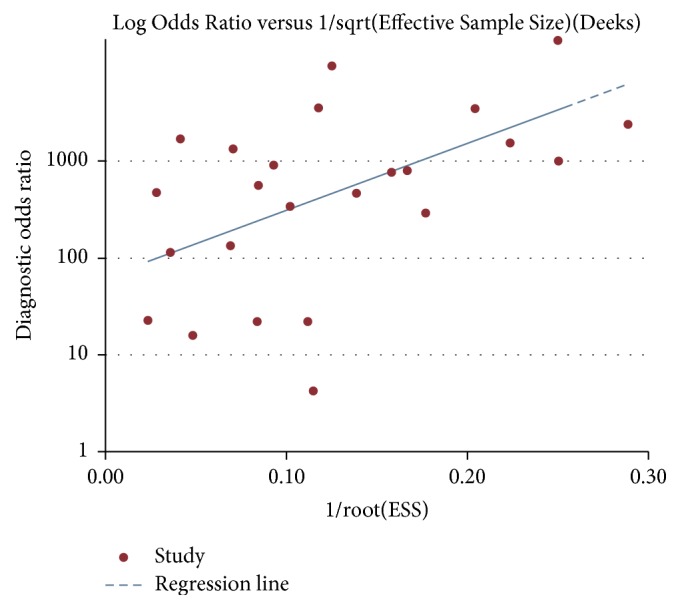
Deek's funnel plot. We found no significant publication bias in the present meta-analysis.

Robustness of pooled results was assessed by sensitivity analysis by deleting one study each time. The analysis indicated that the pooled results were robust. Deek's funnel plot was used to assess publication bias. The funnel plot seemed to be symmetrical (Figure 4).

## 4. Discussion

CHD is a series of heterogeneous disorders that catheter intervention or surgery is mandatory to achieve patients' survival. Clinical examination shows limitation in detecting all forms of CHD [32, 33]. Heart murmurs, one of the hallmarks of CHD, may be misleading or absent due to the reduced ventricular function, prolonged decline of pulmonary vascular resistance, and underlying anatomy. Although prenatal diagnosis is widely applied, a large proportion of CHD neonates are still not diagnosed before being discharged and after birth [34, 35], which may be strengthened by earlier discharge and certain postnatal care [36]. It has been thought that the application of prenatal ultrasound, clinical observation, and physical examination may be sufficient for early diagnosis of CHD [37]. This opinion may be true under specific circumstances; however, the prerequisites possibly do not exist in the majority of hospitals. Thus, a broad consensus that efficient diagnostic tool for CHD is urgently needed has been achieved.

Pulse oximetry is an accurate and noninvasive test for quantification of hypoxaemia. The application of this method for diagnosing CHD is based on the theory that undetectable hypoxaemia in clinic exists in most life-threatening cases. Pulse oximetry screening for CHD has gained more attention over the last decade. It has been demonstrated to be cost-effective and acceptable to the mothers [38, 39]. The existing protocol of pulse oximetry to detect CHD is restricted to 24 to 48 hours of age for neonates in well infant nursery [40].

Our meta-analysis included as many eligible articles as possible via systematic search. These obtained articles were selected carefully according to inclusion criteria. Moreover, the quality of included articles was high. Besides, the results were based on 22 eligible studies involving both Western and Asian countries. Therefore, our results were reliable. The pooled results suggested that combined sensitivity, specificity, and AUC were 0.69 (0.67–0.72), 0.99 (0.99-0.99), and 0.9407, respectively, which is similar to the previous meta-analysis [41].

Among the included articles, the results showed great differences. In the study by Mathur et al., pulse oximetry readings were taken at admission from 950 neonates and the diagnostic sensitivity, specificity, positive predictive value, and negative predictive value were 95.2%, 52.4%, 9.5%, and 99.5%. The diagnostic specificity was poor. Similarly, Hu et al. reported that diagnostic specificity of pulse oximetry screening for CHD was just 44.22%. Meanwhile, Niekerk et al. reported that the diagnostic sensitivity of pulse oximetry was merely 50%, while the specificity was 99.9%. On the contrary, Arlettaz et al. investigated the contribution of pulse oximetry to the early detection of CHD in newborns and found that the sensitivity and specificity were 100% and 99.7%, respectively. In the study of Jones et al., the estimated sensitivity and specificity were 100% and 99.8% of pulse oximetry screening for diagnosing CHD. Thus, the conclusion of our analysis is significant to confirm the diagnostic role of pulse oximetry screening.

However, we must acknowledge that there were limitations in the present meta-analysis. First, cut-off value, diagnosis criteria, target location, and test timing of pulse oximetry were inconsistent among the included studies, which might affect the diagnostic accuracy of pulse oximetry screening. And the significant heterogeneity in the present analysis might result from these variances. Besides, the difference in the severity of CHD also might influence the accuracy of pulse oximetry screening.

## 5. Conclusion

In conclusion, pulse oximetry screening may serve as a valuable diagnostic tool with high accuracy for CHD. The diagnostic sensitivity and specificity are 0.69 (0.67–0.72) and 0.99 (0.99-0.99), respectively.

## Figures and Tables

**Table 1:** Basic information of included studies.

Author	Year	Country	Limb	Test timing, h
Arlettaz et al.	2006	Switzerland	Right or left foot	6–12
Bakr and Habib	2005	Egypt	Right upper and lower limbs	31.7 (average)
de Wahl Granelli et al.	2005	Sweden	Right hand and one foot	12–48
Hoke et al.	2002	America	Right arm and either leg	24 (average)
Koppel et al.	2003	America	—	>24
Richmond et al.	2002	UK	One or other foot	>2
Rosati et al.	2005	Italy	—	72 h (median)
Zhao et al.	2014	China	Both on the right hand and on either foot	6–72
Riede et al.	2010	Germany	Foot	24–72
Mathur et al.	2015	India	Right upper limb and either foot	72 (median)
Hu et al.	2016	China	Right hand and either foot	25 (median)
Jones et al.	2016	UK	—	<24
Ozalkaya et al.	2016	Turkey	Lower and right upper extremity	>24
Jawin et al.	2015	Malaysia	Left foot	20 (median)
Ewer et al.	2011	UK	Right hand and either foot	In the first few hours
Taksande et al.	2013	India	All the four limbs	Within the first 4 hours
van Niekerk et al.	2016	South Africa	—	60 (median)
de-Wahl Granelli et al.	2009	Sweden	Right hand and either foot	38 (median)
Meberg et al.	2008	Norway	Foot	<12
Mo et al.	2015	China	Right hand and either foot	>24
Jia et al.	2016	China	Right hand	24
Lu et al.	2016	China	Right hand and right foot	>24

*Note*. — indicates that the information was not mentioned in the article.
